# Bereavement and Grief Among Employees in an Arab University Setting: A Cross-Sectional Study

**DOI:** 10.7759/cureus.55659

**Published:** 2024-03-06

**Authors:** Randah R Hamadeh, Yousef A Alshammari

**Affiliations:** 1 College of Medicine and Medical Sciences, Arabian Gulf University, Manama, BHR

**Keywords:** educational institution, workplace, death and dying, grief, bereavement

## Abstract

Background: Several mental health outcomes develop following bereavement. Little research has examined bereavement in the workplace and the associated risk factors, particularly in Arab populations.

Objectives: The objectives of this cross-sectional study were to determine the sociodemographic characteristics of bereaved employees, measure the prevalence of their dysfunction, establish the type of closeness and conflict in their relationship with the deceased, determine the available resources to the bereaved, and determine the proportion of bereaved employees who needed help.

Methods: A study was conducted on Arabian Gulf University employees (91) in Bahrain. The revised Two Track Bereavement Questionnaire (TTBQ3-CG11) was utilized to assess bereavement outcomes.

Results: The response rate of the study was 28%. The composition of the study population was as follows: 51.6% males, 37.4% in the age range of 40-49 years, 86.8% married, 39.6% Bahraini, and 51.6% academicians. Over half of the participants had biopsychosocial dysfunction, 35.2% had active relational grief and trauma (ARGT), 36.3% had a conflict with the deceased, and half were close to the deceased. Total TTBQ3-CG11 scores showed that 28.6% of the bereaved had a low score (14-22), 61.5% medium (23-28), and 9.9% high (29 or more), with more females than males in the high category. The majority reported receiving adequate support from the administration and colleagues following their loss.

Conclusion: There is a need to establish bereavement policies and procedures at tertiary educational institutes. This study may inform future policies to advance bereavement services in the educational institutions of the region.

## Introduction

Bereavement is defined as the experience of losing a loved one, where grief constitutes the complex psychobiological response to loss [1]. Multiple health consequences can emerge in the wake of bereavement, with mental health disorders being a major concern [2]. Common psychological consequences include depression, anxiety, stress, and feelings of loneliness. When grief is extended and intense, it can evolve into a condition known as complicated grief, which can impede the natural healing process [3]. Prolonged grief might result in complicated grief that deters healing [1]. It was reported that about 7% of bereaved adults develop complicated grief [4]. There are cultural and gender diversities in the reaction to bereavement and grief, as well as coping strategies [1,3,5]. A study attempting to investigate the gender differences and the effect of bereavement on mental health in China concluded that bereavement had a higher impact on older women compared to men [6]. A Taiwanese post-earthquake study on bereaved men and women found that women were three times more likely to develop post-traumatic stress disorder in comparison to men [7]. Spirituality and religion were found to be generally helpful in coping with the loss of a loved one. However, this varies due to the inconsistency of measuring religion and spirituality [8]. The effects of bereavement are not solely psychologically confined but often translate into physical health complications. The most common self-reported physical symptoms among bereaved spouses are disturbance in sleep (63%), fatigue (54%), problems in concentration (53%), and loss of appetite (43%) [9]. Mortality from all causes was reported to be significantly higher among widowed than non-widowed individuals, in both sexes [10]. A cohort study from Finland that examined mortality after a spouse’s death reported that excess mortality was much higher in shorter periods of bereavement compared to longer periods [11].

Little research has examined bereavement in the workplace and the associated risk factors [12]. It is reported that grief impacts bereaved employees’ work performance, health and safety, and behavior and increases sick leave [13]. Half of the family members whose loved ones passed away in the intensive care unit had problems returning to work and functioning effectively [14]. Parents of children who passed away due to suicide had a tenfold risk of mental illness, with absentees from work exceeding 30 days compared to parents of live children [15]. Hall and colleagues studied the effect of a compassionate community in the workplace and found that the collective response of colleagues and employers had a long-lasting effect on the bereaved person. Conversely, poor or inadequate support can have negative long-term consequences [16]. Gilbert and colleagues suggested a model comprised of four themes: communication, accommodation, recognition, and emotional support (CARE) to follow when bereaved employees return to work. Most of the bereavement leaves are around three days, which implies that bereaved employees return to work while experiencing significant pain [17]. A Canadian study reported that on average, 3.2% of employees in three organizations had taken a bereavement leave consisting of 2.5 days on average per year [18].

To our knowledge, this is the first study in the Eastern Mediterranean region to address bereavement in the workplace and an academic institution. Arabian Gulf University (AGU) students are mostly nationals of Gulf Cooperation Council (GCC) countries while the employees are international. AGU offers its employees three days of leave after the death of a close relative. However, Muslim women employees are given 130 days of paid leave at the death of their spouse. This is in accordance with Islam, where bereaved women are expected to be in waiting for four months and 10 days. The aim of this quality improvement study is to use this data to improve bereavement services at AGU and in institutions in the Eastern Mediterranean region. The objectives of this study were to describe the sociodemographic characteristics of bereaved AGU employees, determine the extent of dysfunction and active grief, ascertain the type of relationship with the deceased, identify the available resources to the bereaved, and estimate the proportion of bereaved employees who needed help.

## Materials and methods

Study design

A cross-sectional study was conducted on all AGU full-time academic and administrative employees (322) during January 2022.

Study participants

The emails of AGU employees were acquired from the Human Resources Department. A group email with a link to a guided response Google document was sent, by one of the investigators followed by three weekly reminders. Due to the delicate nature of the subject, and the anticipated resistance in responding, all employees were considered in the hope of acquiring the maximum response rate possible.

Data sources/measurements

The survey was in English and included sociodemographic characteristics in the first part. The second part had questions related to bereavement, such as bereavement leave, attendance at funerals, and support from the university and colleagues at the time of loss. The final part contained the revised Two Track Bereavement questionnaire for complicated grief, TTBQ3-CG11. It is an update of the validated TTBQ2-CG31 [19] questionnaire for all bereavement cases, including COVID-19 losses. The validity of TTBQ3-CG11 was verified using exploratory factor analysis and the principal component method, followed by an orthogonal ratio with the varimax method for all items. The questionnaire consists of 11 self-reported questions and is rated on a 5-point Likert scale [20]. Track I of the scale explores biopsychosocial dysfunction (DF) and the resources available to the bereaved. Meanwhile, track II explores the bereaved relationship with the deceased using three variables: active relational grief and trauma (ARGT), conflict in the relationship (RC), and close and positive relationship (CPR). According to the TTBQ3-CG11 guidelines, a score of >5 or higher was considered positive for DF, and a score of 7 or more was considered a score requiring clinical attention. ARGT was considered positive if the score was higher than 18 and clinical if it was 23 or higher. A score of 3 or higher in questions related to RC was considered a positive conflict in the relationship. Scores of 4 or higher implied that monitoring was required in relation to CPR-related questions. Needing help was assessed with one question and a reverse score exceeding 4 or more indicated a need for clinical help. Resources available to the bereaved were assessed with one question, whereby reversal scores of 4 or higher were considered clinical. The total sum of scores between 14 and 22 (low) indicated that the bereaved should be followed up at 6 months, and scores between 23 and 28 (medium) indicated that the bereaved should be monitored at three months along with clinical intervention considered. Total scores of 29 or more (high) implied that the bereaved must receive intervention with high priority and be monitored for 1 month.

Ethical approval was obtained from the Research and Ethics Committee, College of Medicine and Medical Sciences, AGU (E33-PI-12-21). A consent form was presented to all study participants at the start of the survey, notifying them of its anonymity and confidentiality and that the survey was voluntary.

Analyses

Descriptive statistics was done to display the characteristics of the study population. DF, resources available, RC, ARGT, and CPR scores were computed based on the TTBQ3-CG11 guidelines. The Chi-square test was used to measure the association between quantitative variables with the respective TTBQ3-CG11 scores. A p-value less than 0.05 was considered statistically significant. IBM SPSS Statistics for Windows, Version 28 (Released 2021; IBM Corp., Armonk, New York, United States) was used for data entry and analysis.

## Results

Demographics

Of the 322 employees, 91 completed the survey yielding a 28% response rate. There were 51.6% males, 37.4% in the age range of 40-49 years, 86.8% married, 39.6% Bahraini, 51.6% academicians, 86.8% living with their own family, 62.6% living in Bahrain for 10 years or more. There were 76.9% of Muslims and 23.1% of other religions with Buddhists (n=14) twice that of Christians (n=7), and 76.9% having much/very much religious observance/belief (Table 1).

**Table 1:** Sociodemographic characteristics of study participants (n=91)

Variable	N (%)
Age group	
20-29 years	4 (4.4)
30-39 years	18 (19.8)
40-49 years	34 (37.4)
50-59 years	17 (18.7)
≥60 years	18 (19.8)
Gender	
Male	47 (51.6)
Female	44 (48.4)
Nationality	
Bahraini	36 (39.6)
Other Arab	21 (23.1)
Non-Arab	34 (37.4)
Educational level	
Bachelors, Medical Degree, and Diploma	24 (26.4)
Master’s degree	27 (29.7)
Doctorate degree	40 (44.0)
Occupation	
Academic	47 (51.6)
Non-Academic	44 (48.4)
Marital status	
Married	79 (86.8)
Other	12(13.2)
Living condition	
With Family	79 (86.8)
Other	12 (13.2)
Years in Bahrain*	
1-4 years	13(14.3)
5-9 years	21 (23.1)
>10 years	57 (62.6)
Religion	
Islam	70 (76.9)
Other	21 (23.1)
Degree of religious observance/belief	
Very Little/Little	6 (6.6)
So-so	15 (16.5)
Much/Very much	70 (76.9)

Loss and resources available

Over half (57.6%) of the employees experienced loss within the past four years, primarily losing an immediate family member, mainly a parent (33.0%). The main cause of death was heart disease (35.3%). Almost two-thirds experienced loss while working at AGU of whom (73.1%) reported receiving support from the university and 60% from their colleagues following their loss. Twenty-two percent of the bereaved requested an extension of their leave at the time of loss. Most (83%) of the bereaved sought help mainly from family and friends (50.8%), religion/spirituality (42.4%), professionals (3.4%), and writing (3.4%). 37.4% believed that the resources were not helpful (Table 2). Females were twice more likely (30.0%) to request an additional leave compared to their male counterparts (14.2%), (p = 0.151). They also relied heavily on family (63.3%) and religion (50%) compared to males (35.5%, and 32.2%, respectively) (p =0.256 and p = 0.322), though the difference was not statistically significant (not in the table).

**Table 2:** Information on the loss, relation to the deceased, and support

Variable	N (%)
Years since death of loved one (n=85)	
1-4 years	49 (57.6)
5-9 years	15 (17.6)
≥10 years	21 (24.7)
Relation to deceased (n=91)	
Parent	30 (33.0)
Sibling	9 (1.1)
Child	5 (5.5)
Spouse	1 (1.1)
Other family relatives	45 (49.5)
Cause of death (n=85)	
Heart disease	30 (35.3)
Lung disease	4 (4.7)
Neurological disease	6 (7.1)
Cancer	9 (10.6)
Chronic illnesses	14 (16.5)
Traumatic accident	6 (7.1)
COVID-19	4 (4.7)
Other	12 (14.1)
Funeral attendance (n=87)	
Attended	51 (58.6)
Did not attend	36 (41.7)
No funeral	4 (4.4)
Received support from university during time of loss (n=52)	
Yes	38 (73.1)
No	14(26.9)
Received support from colleagues during time of loss (n=55)	
Yes	33 (60.0)
No	22 (40.0)

Dysfunction, active grief, and conflict within the bereaved

Dysfunction was noted among 61.5% of the participants, 35.2% had ARGT while 36.3% had conflict in the relationship with the deceased. Dysfunction varied by sociodemographic characteristics, nature of relation with the deceased, and support but none of the variations were statistically significant except for relation with the deceased (p=0.004). The dysfunction among those who lost an immediate family member (76.1%) was higher than for those who had other losses (46.7%). Higher dysfunction (72.2%) was noted among the older employees (≥60 years), males (51.6%) non-Muslims (71.4%), and those who had their loss for 10 years or more (71.4%). Variations in ARGT were statistically significant for religion (p=0.022) and relation to the deceased (p=0.035). 41.4% of Muslims and those who lost an immediate relative (45.7%) had more ARGT. There were borderline differences by nationality (p=0.072) where higher proportions of Bahraini (41.7%) and other Arab (47.6%) had ARGT compared to non-Arabs (20.6%). Conflict relationship with the deceased was statistically significant (p=0.008) by occupation. Half of the nonacademic bereaved employees reported a conflict with the deceased compared to 23.4% of the academic (Table 3).

**Table 3:** Dysfunction, active grief, and conflict among bereaved employees (n=91)

Variable	N (%)	Dysfunction (%)	p-value	Active Grief & Trauma (%)	p-value	Conflict (%)	p-value
Age group							
≤49 years	56 (61.5)	58.9	0.582	28.6	0.215	41.1	0.48
50-59 years	17 (18.7)	58.8		41.2		29.4	
≥60 years	18 (19.8)	72.2		50		27.8	
Gender							
Male	47 (51.6)	63.8	0.642	34	0.817	34	0.649
Female	44 (48.4)	59.1		36.4		38.6	
Nationality							
Bahraini	36 (39.6)	63.9	0.615	41.7	0.072	44.4	0.404
Other Arab	21 (23.1)	52.4		47.6		33.3	
Non-Arab	34 (37.4)	64.7		20.6		29.4	
Educational level							
Bachelors, Medical & Diploma	24 (26.4)	62.5	0.444	20.8	0.125	50	0.444
Master’s	27 (29.7)	70.4		48.1		37	
Doctorate	40 (44.0)	55		35		27.5	
Occupation							
Academic	47 (51.6)	59.6	0.691	31.9	0.502	23.4	0.008
Non-Academic	44 (48.4)	63.6		38.6		50	
Marital status							
Married	79 (86.8)	60.8	0.695	34.2	0.613	36.7	0.821
Other	12(13.2)	66.7		41.7		33.3	
Living condition							
With Family	79 (86.8)	58.3	0.807	36.7	0.429	35.4	0.676
Other	12 (13.2)	62		25		41.7	
Religion							
Islam	70 (76.9)	58.6	0.288	41.4	0.022	38.6	0.403
Other	21 (23.1)	71.4		14.3		28.6	
Degree of religious observance/belief							
Very Little/Little	6 (6.6)	66.7	0.96	16.7	0.209	16.7	0.172
So-so	15 (16.5)	60		20		20	
Much/Very much	70 (76.9)	61.4		40		41.4	
Years since death of loved one *							
1-4 years	49 (53.8)	61.2	0.526	44.9	0.148	36.7	0.949
5-9 years	15 (16.5)	53.3		20		33.3	
≥10 years	21 (23.1)	71.4		28.6		33.3	
Relation to deceased							
Immediate family member	46 (50.5)	76.1	0.004	45.7	0.035	30.4	0.242
Other (family relatives/friends)	45 (49.5)	46.7		24.4		42.2	

Closeness and resources available to the bereaved

Half of the participants had close and positive relationships with the deceased and 37.4% sought resources to help them through their loss. The close and positive relationship had statistically significant variations (<0.005) by age and relationship to the deceased (Table 4). Employees 50 years and above reported more closeness to the deceased (64.7% for the 50-59 years, 66.7% for the 60 and above) compared to those below 50 years (39.3%). Furthermore, closeness to the deceased among those who lost an immediate family member (67.4%) was twice that of other losses (31.1%). There were statistically significant differences among the bereaved who used resources to help them cope with their loss by the educational level and religion. A much lower percentage (16.7%) of those holding less than a graduate degree (37.5%) sought resources than holders of higher degrees (55.6%), (p = 0.0016). In addition, more Muslim employees (44.3%) sought resources to help them cope than non-Muslims (14.3%, p=0.013). Overall, 13.2% (males 11.4%, females, 14.9%) of the bereaved needed help. Almost 20% of those who lost an immediate family member needed help compared to 6.7% for other losses (p=0.069) (Table 4). Furthermore, a higher proportion of those who lost an immediate relative reported that their functioning was reduced (41.3%) in one area or more (work or study; relationship with family or friends; and own health) since the death of their loved one compared to 22.2% for those who had other losses (Table 4).

**Table 4:** Closeness and resources available and needing help in bereaved employees (n=91)

Variable	N (%)	Closeness (%)	p-value	Resources (%)	p-value	Need help (%)	p-value
Age group							
<49 years	56 (61.5)	39.3	0.049	44.6	0.175	14.3	0.925
50-59 years	17 (18.7)	64.7		29.4		11.8	
>60 years	18 (19.8)	66.7		22.2		16.7	
Gender							
Male	47 (51.6)	48.9	0.919	38.3	0.849	14.9	0.619
Female	44 (48.4)	50		36.4		11.4	
Nationality							
Bahraini	36 (39.6)	52.8	0.452	44.4	0.429	8.3	0.487
Other Arab	21 (23.1)	57.1		38.1		19	
Non-Arab	34 (37.4)	41.2		29.4		14.7	
Educational level							
Bachelors, Medical & Diploma Degrees	24 (26.4)	37.5	0.181	16.7	0.0016	12.5	0.957
Master’s	27 (29.7)	44		55.6		14.8	
Doctorate	40 (44.0)	60		37.5		12.5	
Occupation							
Academic	47 (51.6)	53.2	0.461	34	0.499	17	0.264
Non-Academic	44 (48.4)	45.5		40.9		9.1	
Marital status							
Married	79 (86.8)	48.1	0.509	38	0.757	13.9	0.594
Other	12(13.2)	58.3		33.3		8.3	
Living condition							
With Family	79 (86.8)	49.4	0.967	40.5	0.112	12.7	0.702
Other	12 (13.2)	50		16.7		16.7	
Religion (n=91)							
Islam	70 (76.9)	50	0.848	44.3	0.013	11.4	0.365
Other	21 (23.1)	47.6		14.3		19	
Degree of religious observance/belief							
Very Little/Little	6 (6.6)	50	0.945	33.3	0.291	33.3	0.187
So-so	15 (16.5)	53.3		20		20	
Much/Very much	70 (76.9)	48.6		41.4		10	
Years since death of loved one *							
1-4 years	49 (53.8)	49	0.485	32.7	0.704	10.2	0.48
5-9 years	15 (16.5)	66.7		33.3		20	
≥10 years	21 (23.1)	52.4		42.9		19	
Relation to deceased							
Immediate family member	46 (50.5)	67.4	0.001	32.6	0.343	19.6	0.069
Other family relatives	45 (49.5)	31.1		42.2		6.7	

Bereavement scores

The total TTBQ3-CG11 scores (Table 5) showed that 28.6% of the bereaved employees had a low score, 61.5% medium, and 9.9% high with more females (13.6%) than males (6.4%) in the high category. High total TTBQ3-CG11 scores were among those 50-59 years old (23.5%), other Arab (19.0%), females (13.6%), those who lost an immediate family member (13.0%), Muslims (11.4%), those with a much/very much religious observance (11.4%), and those who experienced loss for five years or more (9.4%).

**Table 5:** Total TTBQ scores in bereaved employees (n=91)

Variable	N (%)	Low / Follow-up at 6 months (%)	Medium / Monitor at 3 months (%)	High / Intervention priority (%)	p-value
Age group					
≤49 years	56 (61.5)	23.2	71.4	5.4	0.03
50-59 years	17 (18.7)	47.1	29.4	23.5	
≥60 years	18 (19.8)	27.8	61.1	11.1	
Gender					
Male	47 (51.6)	29.8	63.8	6.4	0.511
Female	44 (48.4)	27.3	59.1	13.6	
Nationality					
Bahraini	36 (39.6)	25	66.7	8.3	0.321
Other Arab	21 (23.0)	19	61.9	19	
Non-Arab	34 (37.4)	38.2	55.9	5.9	
Educational level					
Bachelors, Medical Degree, and Diploma	24 (26.4)	37.5	58.3	4.2	0.721
Master’s degree	27 (29.7)	25.9	63	11.1	
Doctorate degree	40 (44.0)	25	62.5	12.5	
Occupation					
Academic	47 (51.6)	29.8	59.6	10.6	0.92
Non-Academic	44 (48.4)	27.3	63.6	9.1	
Marital status					
Married	79 (86.8)	29.1	59.6	11.4	0.399
Other	12(13.2)	25	75	0	
Living condition					
With Family	79 (86.8)	27.8	60.8	11.4	0.464
Other	12 (13.2)	33.3	66.7	0	
Religion					
Islam	70 (76.9)	22.9	65.7	11.4	0.08
Other	21 (23.1)	47.6	47.6	4.8	
Degree of religious observance/belief					
Very Little/Little	6 (6.6)	33.3	66.7	0	0.439
So-so	15 (16.5)	46.7	46.7	6.7	
Much/Very much	70 (76.9)	24.3	64.3	11.4	
Years since death of loved one *					
1-4 years	49 (53.8)	32.7	59.2	8.2	0.583
5-9 years	15 (16.5)	26.7	60	13.1	
≥10 years	21 (23.1)	14.3	71.4	14.3	
Relation to deceased					
Immediate family member	46 (50.5)	19.6	67.4	13	0.129
Other (family relatives/friends)	45(49.5)	37.8	55.6	6.7	

## Discussion

This study is one of the few studies in the Eastern Mediterranean region that examined bereaved employees at the workplace, measured dysfunction, and assessed complicated grief in a higher education setting. Half of the bereaved employees lost an immediate family member, and over half experienced the loss within four years from the date of the survey. As in the literature, family and friends were the main sources of support in the grief journey of the employees [21,22] with more females relying on family, as has been reported elsewhere [21,23]. It is not surprising that only 3.4% sought professional help, which reflects a reluctance to seek professional help due to cultural views on bereavement. More women sought help than men although men were more in need of help. It is worth noting that men are less likely to seek external help than women, particularly in mental health [24].

Sixty-two percent of the bereaved employees exhibited DF, 35.2% ARGT, and 36.3% had conflict in their relationship with the deceased. The death of an immediate family member was associated with an increased risk of dysfunction, and ARGT. There were gender differences in the total TTBQ scores, with more females (13.6%) requiring priority intervention and monitoring after a month than males (6.4%). Females are at a higher risk of depressive disorders, anxiety disorders, and eating disorders than males [25]. In a study of bereaved US Chinese, females had poorer outcomes for depression, anxiety, stress, and loneliness [3]. A recent study that assessed the impact of COVID-19 on the AGU population showed a gender disparity in anxiety, where females (20%) were five times more likely than males (3.4%) to have severe anxiety [26].

Being Muslim was associated with an increased risk of ARGT. In addition, more Muslims required medium-to-moderate intervention than non-Muslims. This finding could be explained by the fact that Islamic beliefs and rituals encourage the remembrance of the bereaved, as Muslims are expected to take comfort from prayers and praying for the deceased. Muslims believe that God is aware of their sorrow and endurance and that they should get along with their lives until they reunite with their loved ones after their death [27]. Moreover, the cultural influence on mental health-seeking behavior in the region, particularly following bereavement, can exacerbate the effect of loss.

Most of the participants received support from the university administration (73.1%) and from their colleagues (60%) during the time of loss. Most of the bereaved employees who experienced loss at AGU did not request additional leave following the death of a loved one. Of those who requested additional leave, 84.6% lost an immediate family member and were mostly female. Going back to work after the loss of a loved one is very challenging for the bereaved, particularly for those who lost a child, a young loved one, or special death circumstances such as an accident, suicide, or violence [28]. A Canadian study showed that mothers had longer grief reactions than fathers following the death of a child and were less work-focused [29].

The university has policies regarding the number of days for bereavement leave, but there are no policies to accommodate the bereaved workers upon their return to work. The literature shows that organizations should be proactive and prepared for such inevitable events [21,29].

The CARE model has been suggested to support bereaved employees, and others have suggested the development of specific policies [17]. We recommend that AGU follow the CARE model to support the bereaved employees when they resume work. The Human Resources Office may contact the bereaved employees and ask them if they need more days of leave, particularly if the deceased is a child, grandchild, or young relative, or if the death was sudden. Furthermore, the counseling unit must be trained in bereavement counseling to assist the bereaved employees after returning from bereavement leave and assist them in seeking professional help. In addition, heads of departments should be accommodating to the specific needs of bereaved employees by being flexible and considerate. It might be helpful to ask employees who experienced losses in the past to support the newly bereaved in dealing with their loss.

Strengths and limitations

To our knowledge, this is the first study in the Arabian Gulf region exploring grief and loss in a university setting, addressing a novel and sensitive topic. The study uncovers the effect of grief on employees. It is also the first to apply the revised TTBQ3-CG11 in the GCC countries. However, the study has several limitations. The low response rate (28%) despite the three reminders was unfortunate but can be partly explained by the hesitancy of the employees to respond to a survey with a delicate and personal topic. Thus, the findings of the study can be considered preliminary as they have implications on the generalizability of the findings. Another limitation is the inability to sample due to the anticipated hesitancy of participation thus hoping to get the largest number of respondents possible. Lastly, the inability to perform multivariable analysis due to the small size of the study population and having three TTBQ3-CG11 categories in each sub-scale did not allow examining for potential confounding factors. Despite the above limitations, this study fills a gap in the current information on bereavement at the workplace and has particular significance in the region.

## Conclusions

Almost two-thirds of the bereaved employees exhibited biopsychosocial dysfunction and over one-third ARGT. Half of the bereaved lost an immediate family member, and over half experienced the loss within four years; 13.2% of the bereaved needed help, and 9.9% required priority intervention. Although the majority reported that they received adequate support from the university administration and colleagues following their loss, there is a need to suggest having bereavement policies and procedures at the university. This study can be considered preliminary and underscores the need to have further research in the region. A large-scale regional multi-center study in the future can fill the gaps in understanding the implications of bereavement at the workplace further.
